# Stimulating the development of orphan (and other) vaccines.

**DOI:** 10.3201/eid0506.990617

**Published:** 1999

**Authors:** B Schwartz, N R Rabinovich

**Affiliations:** Centers for Disease Control and Prevention, Atlanta, GA, USA.


### Stimulating the Development of Orphan (and Other) Vaccines

Drs. Lang and Wood (this issue, pp. 749-756) highlight factors that affect vaccine development decisions at large pharmaceutical companies and suggest measures to make development of orphan vaccines more attractive. Because of the importance of economic assessment in corporate decision-making, development of vaccines for rare diseases is usually problematic. Exceptions may include vaccines for potential bioterrorism agents (the government may support development and production) and therapeutic vaccines for chronic or deadly diseases (the price of a vaccine could be high, commensurate with the cost of therapy). Of seven vaccines defined by the Institute of Medicine as being “most favorable” for development, three were therapeutic vaccines (for diabetes mellitus, rheumatoid arthritis, and multiple sclerosis) (1).

In the developing world, price has been a major impediment to the introduction of new vaccines. Whether this reflects limitations in ability or willingness to pay, the end result is that a company could not expect sales in developing countries to provide the desired return on investment (2). Clearly, novel solutions are needed if vaccines that could save millions of children’s lives are to be used effectively. Support for vaccination from the Bill and Melinda Gates Foundation and the promotion of vaccines as an acceptable component of bilateral loans by the World Bank may begin shifting the balance between market imperatives and public health needs.

Drs. Lang and Wood propose a package of incentives that may help promote development of orphan vaccines by major manufacturers. But will these measures be enough to alter vaccine development priorities? Lowering the risks or costs of vaccine development may be much less important than increasing the potential for profit. The vaccine development pipeline is full of products that will never come to market, not because they cost more to develop but because the company projects insufficient profit from their eventual use. Promoting a greater appreciation of the benefits of prevention in both developing and industrialized countries and enhancing the size of the market and the willingness to pay will likely have a greater impact on investment decisions than an incremental decrease in vaccine development costs.

If large manufacturers shift vaccine development priorities on the basis of incentives and other measures so that the total number of products brought to market is not increased but one set of priorities is substituted for another, the overall impact on disease prevention may be not change. The greatest increase in disease prevention and in the development of orphan vaccines would occur by increasing the total number of vaccines produced. The therapeutics industry differs from the vaccine industry in that it includes a substantially greater number of players that can bring a new product to market. In the United States currently, 194 drugs and biologics have been brought to market as orphan products, but none are vaccines. Thus, incentives that draw new companies to invest in vaccine development may be extremely useful for the development of orphan vaccines.

Vaccines prevent more than 3.2 million deaths per year (3). Developments in biotechnology have created the promise of prevention for many more infectious and chronic diseases (4). Realizing this promise will require bringing to licensure more of the vaccines now in development. Finally, our credibility in designating disease areas as priorities for vaccine development rests on our ability to use the new vaccines already in hand.

**Benjamin Schwartz* and N. Regina Rabinovich†**
*Centers for Disease Control and Prevention, Atlanta, GA, USA; †National Institute of Allergy and Infectious Diseases, National Institutes of Health, Bethesda, MD, USA

### References

1. Institute of Medicine. Vaccines for the 21st century: a tool for decision-making. Washington: National Academy Press, 1999.
2. Hausdorf WP. Prospects for the use of new vaccines in developing countries: cost is not the only impediment. Vaccine 1996;14:1179-86.
3. World Health Organization, United Nations Children’s Fund. State of the world’s vaccines and immunization. Geneva, Switzerland: The Organization, 1996.
4. Jordan Report. Accelerated development of vaccines 1998. Washington: National Institute of Allergy and Infectious Diseases, 1998.

